# Development and Validation of a Prediction Model for Elevated Arterial Stiffness in Chinese Patients With Diabetes Using Machine Learning

**DOI:** 10.3389/fphys.2021.714195

**Published:** 2021-08-23

**Authors:** Qingqing Li, Wenhui Xie, Liping Li, Lijing Wang, Qinyi You, Lu Chen, Jing Li, Yilang Ke, Jun Fang, Libin Liu, Huashan Hong

**Affiliations:** ^1^Fujian Key Laboratory of Vascular Aging, Department of Geriatrics, Department of Cardiology, Department of Cardiac Surgery, Fujian Heart Disease Center, Fujian Institute of Geriatrics, Fujian Medical University Union Hospital, Fuzhou, China; ^2^Department of Endocrinology, Fujian Medical University Union Hospital, Fuzhou, China

**Keywords:** arterial stiffness, LASSO, machine learning, gradient boosting, web tool

## Abstract

**Background:**

Arterial stiffness assessed by pulse wave velocity is a major risk factor for cardiovascular diseases. The incidence of cardiovascular events remains high in diabetics. However, a clinical prediction model for elevated arterial stiffness using machine learning to identify subjects consequently at higher risk remains to be developed.

**Methods:**

Least absolute shrinkage and selection operator and support vector machine-recursive feature elimination were used for feature selection. Four machine learning algorithms were used to construct a prediction model, and their performance was compared based on the area under the receiver operating characteristic curve metric in a discovery dataset (*n* = 760). The model with the best performance was selected and validated in an independent dataset (*n* = 912) from the Dryad Digital Repository (https://doi.org/10.5061/dryad.m484p). To apply our model to clinical practice, we built a free and user-friendly web online tool.

**Results:**

The predictive model includes the predictors: age, systolic blood pressure, diastolic blood pressure, and body mass index. In the discovery cohort, the gradient boosting-based model outperformed other methods in the elevated arterial stiffness prediction. In the validation cohort, the gradient boosting model showed a good discrimination capacity. A cutoff value of 0.46 for the elevated arterial stiffness risk score in the gradient boosting model resulted in a good specificity (0.813 in the discovery data and 0.761 in the validation data) and sensitivity (0.875 and 0.738, respectively) trade-off points.

**Conclusion:**

The gradient boosting-based prediction system presents a good classification in elevated arterial stiffness prediction. The web online tool makes our gradient boosting-based model easily accessible for further clinical studies and utilization.

## Introduction

Cardiovascular disease (CVD) remains the leading cause of death worldwide (Zhao et al., 2019). Arterial stiffness is a vascular measure that has been reported to predict cardiovascular events (Munakata, 2014). It is the common pathological basis for CVD, such as hypertension, atherosclerosis, and stroke, and has been linked to the aging cardiovascular continuum (O’Rourke et al., 2010; Donato et al., 2018; Zhang and Hong, 2019). Arterial stiffness increases with vascular aging due to gradual loss of arterial elasticity and is accelerated by conditions that increase cardiovascular risk, including diabetes mellitus (DM) (Horton et al., 2021). Clinically, brachial-ankle pulse wave velocity (baPWV) is a unique measure of systemic arterial stiffness (Munakata, 2014). Individuals with baPWV > 1400 cm/s are considered to have vascular aging (VA) (Sang et al., 2020), indicating a moderate risk level of the Framingham Risk Score (Yamashina et al., 2003) and increased risk of hypertension (Tomiyama et al., 2009; Wang Y. et al., 2016; Chen et al., 2017; Yang et al., 2018). Although considerable effort has been made to reduce the CVD risk, the number of individuals with elevated arterial stiffness risk for CVD is large and the application of the baPWV measurement is limited. Thus, the necessity of a simple and convenient clinical tool to assess elevated arterial stiffness in daily clinical practice is highlighted.

The development of a risk scoring system based on simple predictors, i.e., clinical data, is an important step toward the monitoring and diagnosis of elevated arterial stiffness. SAGE based on a multiple logistic regression (LG) was introduced as a method to predict elevated arterial stiffness (Xaplanteris et al., 2019). However, the LG-based approach fails to consider the complex non-linear interactions between variables, which can be captured by more sophisticated model algorithms, thus improving the accuracy of risk prediction. Recently, machine learning has been widely applied to the development of clinical tools for disease diagnosis (Rajkomar et al., 2019; de Gonzalo-Calvo et al., 2020; Zhang Z. et al., 2021). Unlike the traditional LG-based approach, machine learning can recognize hidden patterns and non-linear interactions in complex data, allowing for a better assessment of clinical outcomes (Myszczynska et al., 2020).

In this study, to our knowledge, we have developed the first machine learning-based clinical scoring system for elevated arterial stiffness in patients with diabetes, validating the model in an independent dataset from a Japanese cohort. We have also developed a user-friendly web application using this risk scoring system, allowing for further study and application of this system.

## Materials and Methods

### Patients

The discovery dataset included a total of 760 patients recruited from Fujian Medical University Union Hospital (Fujian, China) from April 2017 to January 2019. The inclusion criteria were as follows: patients diagnosed with DM (American Diabetes Association, 2019), older than 18 years, first visited our clinic, and underwent a baPWV test. The exclusion criteria were as follows: patients with an ankle-brachial index (ABI) less than 0.9 (Ato, 2018); diagnosis of severe arrhythmia, pulmonary, renal, rheumatic diseases, heart valve disease, aortopathy, and myocarditis; and antibiotic and probiotic usage in the past 3 months. The study was conducted in accordance with the Declaration of Helsinki (as revised in 2013). The study was approved by the Medical Faculty of Fujian Medical University Union Hospital Ethics Committee (NO.: 2020KY031) and individual consent for this retrospective analysis was waived.

The validation dataset from an existing study from the Dryad Digital Repository^1^ (Fukuda et al., 2014b) was used to further evaluate the performance of the predictive model. A total of 912 patients from Murakami Memorial Hospital in Japan from March 2004 to December 2012 were recruited in this study. Detailed information about this cohort is described in the original study publication (Fukuda et al., 2014a).

### Assessment of Elevated Arterial Stiffness and Measurement of Other Covariants

The automatic artery stiffness tester BP203RPE-II (VP-1000; Omron, Kyoto, Japan) was used to measure baPWV, blood pressure, and ABI. The patients were divided into two groups: baPWV ≥ 1,400 cm/s as the elevated arterial stiffness (EAS) group and baPWV < 1,400 cm/s as the non-EAS group (non-EAS).

A standardized questionnaire regarding demographic characteristics, blood test indicators, arterial elasticity indicators, hemodynamic parameters, echocardiographic parameters, and carotid artery ultrasound parameters was administered by the same trained team of interviewers. The body mass index (BMI) was based on the height and weight: BMI (kg/m^2^) = weight (kg)/height^2^ (m^2^). The estimated glomerular filtration rate (eGFR) was calculated according to the CKD-EPI formula (Biljak et al., 2017). The ascending aortic diameter (AO) and other parameters were measured *via* echocardiography according to the American Society of Echocardiography guidelines (Mitchell et al., 2019). The internal diameter of the common carotid artery and other parameters were measured by carotid vascular ultrasound. Alcohol consumption was categorized into two groups: no alcohol and > 30 g/week beginning at least 1 year after drinking (Yang et al., 2010). The smoking status was classified into two groups: non-smoker and current smoker (continuously smoking one or more cigarettes a day for at least 6 months) (Qian et al., 2010). Postmenopausal state was defined as amenorrhea for 12 consecutive months, excluding other pathological or physiological causes (Hamaguchi et al., 2012). Coronary heart disease was diagnosed according to the European Society of Cardiology (ESC) guidelines (Taylor, 2013). Hypertension was diagnosed according to the ESC guidelines (Williams et al., 2018). Diabetes was diagnosed according to the American Diabetes Association guidelines (American Diabetes Association, 2019). Carotid artery plaque was diagnosed according to the European Mannheim consensus (Touboul et al., 2012). Carotid intima-media thickness (CIMT) was evaluated using ultrasound, and CIMT > 1 mm signified the thickening of carotid intima (Guo et al., 2020).

### Feature Selection

Least absolute shrinkage and selection operator (LASSO) is a compression estimation algorithm, which adds a penalty parameter to least squares regression to compress the estimated variables, thereby improving the prediction accuracy and interpretation of a model (Cecelja et al., 2020; Zhang K. et al., 2021). Thus, we used LASSO to select candidate variables. In addition, a support vector machine-recursive feature elimination (SVM-RFE) analysis was performed for variable selection (Wang L. et al., 2016). Finally, we combined variables from either the LASSO or SVM-RFE algorithm and then selected variables that are easily available in clinical practice for subsequent model development. LASSO and SVM-RFE were performed using glmnet (version 3.0−2) and e1071 (version 1.7−3) R packages, respectively.

### Machine Learning and Parameter Tuning

Accurate prediction of EAS is important for clinical treatment decisions and can avoid excessive medical treatment caused by false-positive prediction. Thus, in this study, we aimed to achieve a simple and high-accuracy predictive model. Machine learning algorithms, including decision tree (DT), support vector machine (SVM), random forest (RF), and gradient boosting (GB), were used to construct the model, and then their performances were compared to determine the best model.

Decision tree is a tree structure model that consists of a root node and several internal nodes and leaf nodes. The root node contains all samples, each internal node represents a decision point corresponding to a single attribute, and each leaf represents a single class label (Podgorelec et al., 2002; Krzywinski and Altman, 2017). The sample was classified based on the structure of the DT model level by level. Given that DT has a high degree of transparency and is not affected by data scaling, we first used DT to construct the model. Although DT can provide a complete decision-making process for clinical problems, it often suffers from overfitting, which increases the complexity of the model and may result in poor performance on generalization. Thus, the second algorithm, SVM, with excellent generalization capability, is also used to construct an optimal classification hyperplane in an N-dimensional feature space (N: the number of features) to separate the two classes of data points. SVM is a supervised learning method based on the principle of structural risk minimization for classification prediction and non-linear regression (Nedaie and Najafi, 2018). Finally, ensemble learning methods including RF and GB, which aim to reduce the variance in models and further improve the accuracy of predictions by combining multiple models instead of using a single model, were used to develop the models. The RF model is based on the DT method, which parallelly combines a large number of DTs using bootstrap resampling to generate a model with a lower variance and better generalization than a single DT (Friedman, 2001). GB goes one step further, improving performance over iterations rather than averaging predictive results from all DTs in an RF (Friedman, 2001). GB generates a new DT based on previous DTs by reducing prediction errors when blended with previous ones.

To obtain optimal hyperparameters, the area under the receiver operating characteristic curve (AUROC) was evaluated based on a 10-fold cross-validation with different parameters in the discovery cohort. We tuned the complexity parameter for the DT model, the ntree and mtry parameters for the RF model, and multiple parameters (interaction.depth, n.tree, shrinkage, and n.minobsinnode) for the GB model. The SVM, DT, RF, and GB models were constructed using svm (version 1.4.0), rpart (version 4.1−15), randomForest (version 4.6−14), and gbm (version 2.1.5) R packages, respectively. *p* < 0.05 indicates a statistically significant difference.

### Assessment of the Model Performance and Model Validation

The discovery data were randomly split into two groups 100 times: training data (70%) and testing data (30%). Each time, we first developed the four different machine learning models on the training data based on the previous tuning parameters. We then calculated the AUROC and area under the precision-recall curve (AUPRC) of the four machine learning algorithms on the testing data. Finally, we compared the values of AUROC and AUPRC from the four models to determine which model performed best. After selecting the best-performing model as the final model, we constructed the GB model using the full discovery data. Youden’s index was calculated to determine the best cutoff value of the GB model. To further validate the classification capacity of the GB model, we applied our trained GB model on an independent validation cohort. The ROC and PRC were analyzed using pROC (version 1.16.2) and PRROC (version 1.3.1) R packages, respectively.

### Web Application Development

To develop a web application for EAS assessment that is applicable in daily clinical practice, we designed a web-based tool, an EAS predictor, allowing access to our final trained model. Specifically, we used front-end development technologies, Node.js (v12.14.0), React (v16.13.1), and Ant Design (v4.5.4), to simplify the development process. RestRserve (v0.3.0) back-end development technology was used to load the final trained model. Data required for prediction were received by the model using the TCP/IP method, and then the predictive result was returned. This web tool is hosted on our server, which is freely accessible *via* http://vascularagingpredictor.top/.

### Statistical Analysis

The Kolmogorov–Smirnov (K–S) test was used to assess the normality of data. Continuous data with a normal distribution are presented as mean values ± standard deviation (SD), whereas continuous data with non-normal distribution are presented as median values (quartile). Student’s *t*-test was used for the comparison of continuous data following a normal distribution, and Mann–Whitney U test was used for the comparison of data with a non-normal distribution. Categorical data are presented as frequency (percentage), and comparisons between two groups were performed using the χ2 test or Fisher’s exact test (if theoretical frequency *T* < 5). The above statistical analysis was performed using R software 3.6.2^2^.

## Results

### Subject Characteristics

This study enrolled 760 subjects with a mean age of 56 ± 12 years (60.1% male, 39.9% female). Based on the dividing value of 1,400 cm/s of baPWV, the subjects were divided into two groups: non-EAS and EAS. The complete data of patients include demographic information, chemistry indicators, diseases, hemodynamic parameters, and echocardiographic and carotid artery ultrasound parameters in each group (Table 1 and Supplementary Tables 1–3). A total of 230 patients with a mean age ± SD of 48 ± 13 (66.5% male, 33.5% female) are in the non-EAS group, whereas a total of 530 patients with age of 60 ± 9 (57.4% male, 42.6% female) are in the EAS group. Significant differences in baPWV values were observed between the non-EAS and EAS groups.

**TABLE 1:** Clinical characteristics of the patients.

		Total	Non-EAS	EAS	*P*
*n*		760	230 (30.26%)	530 (69.74%)	–
Male, *n*(%)		457 (60.13%)	153 (66.52%)	304 (57.36%)	0.018
Age, years		56.39 ± 12.07	47.77 ± 13.13	60.14 ± 9.37	0.000
Age ≥ 65 years, *n*(%)		224 (29.47%)	23 (10.00%)	201 (26.48%)	0.000
Height, cm		163.96 ± 8.94	166.43 ± 9.32	162.89 ± 8.56	0.000
Weight, kg		65.69 ± 12.34	67.09 ± 13.12	65.08 ± 11.94	0.039
BMI, kg/m^2^		24.35 ± 3.73	24.14 ± 3.97	24.44 ± 3.62	0.306
Waist, cm		88.36 ± 9.94	87.04 ± 9.95	88.94 ± 9.90	0.015
Postmenopausal (female), *n*(%)		244 (80.53%)	36 (46.75%)	208 (92.04%)	0.000
Current smoker, *n*(%)		172 (22.63%)	25 (10.87%)	147 (27.74%)	0.000
Current drinker, *n*(%)		182 (23.94%)	40 (17.39%)	142 (26.79%)	0.005
Comorbidity, *n*(%)		–	–	–	–
	Hypertension	352 (46.32%)	51 (22.17%)	301 (56.79%)	0.000
	Coronary heart disease	47 (6.18%)	8 (3.48%)	39 (7.36%)	0.041
	Ischemic stroke	25 (3.29%)	3 (1.30%)	22 4.15%)	0.043
Type of diabetes, *n*(%)		–	–	–	–
	Type 1	64 (8.42%)	37 (16.09%)	27 (5.09%)	–
	Type 2	689 (90.66%)	188 (81.74%)	501 (94.53%)	–
	Other type	7 (0.92%)	5 (2.17%)	2 (0.37%)	0.000
Complication of diabetes, *n*(%)		–	–	–	–
	Nephropathy	150 (30.8%)	13 (9.77%)	137 (38.7%)	0.000
	Retinopathy	167 (34.29%)	26 (19.55%)	141 (39.83%)	0.000
	Peripheral neuropathy	340 (69.82%)	75 (56.39%)	265 (74.86%)	0.000
Visceral fat area, cm^2^		77.71 ± 43.65	67.45 ± 43.67	81.49 ± 43.10	0.002
Atherosclerosis, *n*(%)		–	–	–	–
	Carotid atherosclerosis	304 (66.38%)	56 (45.53%)	248 (74.03%)	0.000
	Lower extremity atherosclerosis	39 (8.14%)	7 (5.47%)	32 (9.12%)	0.196
Inspection index		–	–	–	–
Leukocyte, X10^ 9/L		6.37 ± 1.74	6.15 ± 1.65	6.44 ± 1.77	0.100
Neutrophils, X10^ 9/L		3.93 ± 1.50	3.58 ± 1.36	4.06 ± 1.53	0.002
Lymphocytes, X10^ 9/L		1.88 ± 0.62	2.02 ± 0.66	1.83 ± 0.60	0.003
Neutrophils/Lymphocytes		2.32 ± 1.29	1.94 ± 0.93	2.47 ± 1.37	0.000
Monocytes, X10^ 9/L		0.39 ± 0.18	0.39 ± 0.12	0.39 ± 0.20	0.708
RDW-SD		40.99 ± 3.76	40.77 ± 4.60	41.07 ± 3.39	0.429
RDW-CV		12.7 ± 1.37	12.69 ± 1.61	12.71 ± 1.27	0.903
Platelet, X10^ 9/L		232.11 ± 71.74	232.42 ± 69.07	231.99 ± 72.8	0.953
PDW, %		12.74 ± 2.34	12.84 ± 2.26	12.70 ± 2.37	0.555
MPV, fl		10.54 ± 1.64	10.43 ± 1.13	10.57 ± 1.79	0.402
Fasting plasma glucose, mmol/L		9.15 ± 4.02	8.94 ± 4.29	9.24 ± 3.91	0.349
ALT, IU/L		27.09 ± 54.71	27.17 ± 30.77	27.06 ± 62.33	0.980
AST, IU/L		37.8 ± 80.77	38.56 ± 83.9	37.47 ± 79.45	0.865
ALP, IU/L		76.01 ± 30.77	77.59 ± 40.98	75.32 ± 25.09	0.437
γ-GT, IU/L		45.83 ± 122.27	48.63 ± 115.36	44.62 ± 125.23	0.678
ALB, g/L		39.18 ± 5.09	39.37 ± 5.11	39.09 ± 5.08	0.485
BUN, mmol/L		5.75 ± 2.78	5.13 ± 1.72	6.02 ± 3.09	0.000
Cr, μmol/L		72.81 ± 32.46	66.25 ± 22.61	75.66 ± 35.54	0.000
eGFR, ml/min/1.73 m^2^		95.31 ± 28.26	108.98 ± 22.67	89.37 ± 28.41	0.000
Stage of CKD, *n*(%)		–	–	–	–
	1	493 (64.87%)	186 (80.87%)	307 (57.92%)	–
	2	181 (23.82%)	39 (16.96%)	142 (26.79%)	–
	3	62 (8.16%)	4 (1.74%)	58 (10.94%)	–
	4	18 (2.37%)	1 (0.43%)	17 (3.21%)	–
	5	6 (0.79%)	0 (0.00%)	6 (1.13%)	0.000
UA, umol/L		336.05 ± 107.19	332.5 ± 108.39	337.6 ± 106.73	0.548
TG, mmol/L		2.96 ± 5.53	3.00 ± 5.87	2.94 ± 5.38	0.906
TC, mmol/L		5.02 ± 1.86	5.02 ± 1.72	5.02 ± 1.92	0.991
HDL-C, mmol/L		1.18 ± 0.43	1.17 ± 0.46	1.18 ± 0.41	0.880
LDL-C, mmol/L		3.02 ± 1.14	3.04 ± 1.08	3.01 ± 1.17	0.666
LDH, IU/L		183.8 ± 46.76	167.42 ± 42.04	189.86 ± 47.01	0.000
CK, IU/L		101.21 ± 86.17	100.86 ± 108.6	101.34 ± 76.45	0.957
CKMB, IU/L		16.14 ± 7.27	16.01 ± 5.51	16.19 ± 7.83	0.812
CRP, mg/L		6.19 ± 15.44	4.70 ± 13.29	6.74 ± 16.14	0.205
TSH, mI/UL		1.99 ± 1.78	1.97 ± 1.59	1.99 ± 1.85	0.896
FT3, pmol/L		5.16 ± 1.53	5.41 ± 1.93	5.07 ± 1.34	0.030
FT4, pmol/L		12.69 ± 5.20	13.12 ± 5.91	12.53 ± 4.90	0.267

*BMI, Body mass index; RDW, Red blood Cell distribution width; PDW, Platelet volume distribution width; MPV, Mean platelet volume; ALT, Alanine aminotransferase; AST, Aspartate aminotransferase; ALP, Alkaline phosphatase; γ-GT, γ-glutamyl transpeptidase; ALB, Albumin; BUN, Blood urea nitrogen; Cr, Creatinine; eGFR, Estimated glomerular filtration rate; UA, Uric acid; TG, Triacylglycerol; TC, Total cholesterol; HDL-C, High density lipoprotein cholesterol; LDL-C, Low density lipoprotein cholesterol; LDH, Lactate dehydrogenase; CK, Creatine Kinase; CKMB, Creatine Kinase MB Subtype; CRP, High sensitivity C-reactive protein; TSH, thyrotropin; FT3, free triiodothyronine; FT4, free thyroxin.*

### Feature Selection

**FIGURE 1:**
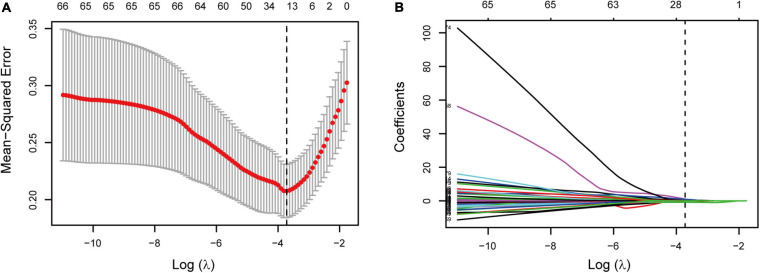
Feature selection based on the LASSO binary logistic regression analysis. **(A)** Optional lambda (λ) value of 0.024 with log(λ) of −3.72 was obtained based on a 10-fold cross-validation and minimum criteria. Dotted vertical line shows the optional λ value. **(B)** LASSO coefficient profiles of 15 features. Vertical line shows the optional λ value that resulted in 15 features with non-zero coefficients.

Two different algorithms, LASSO and SVM-RFE, were applied to select the most significant features for classifying individuals with normal (<1,400 cm/s) or abnormally elevated baPWV (≥1,400 cm/s). First, all features (a total of 99 variables) were included in the LASSO regression analysis and narrowed down to 15 features with non-zero β coefficients in the LASSO regression model (Figures 1A,B and Supplementary Table 4). Second, SVM-RFE was analyzed to select the top 15 important features (Supplementary Table 5). We combined features from either the LASSO or SVM-RFE algorithm, and then we further selected four variables (age, SBP, DBP, and BMI) that are easily available in clinical practice for subsequent model construction.

### Parameter Optimization and Model Selection

Before the model construction using the full discovery dataset, we first tuned the parameters of the model based on a 10-fold cross-validation. We found that when the complexity parameter of the DT model was set as 0, the model achieved the highest AUC value, whereas when the RF ntree = 1,000 and mtry = 2, the model achieved the best performance. For the GB model, the best performance was obtained when interaction.depth = 2, n.trees = 400, shrinkage = 0.02, and n.minobsinnode = 5 (Supplementary Figures 1A–C).

**FIGURE 2:**
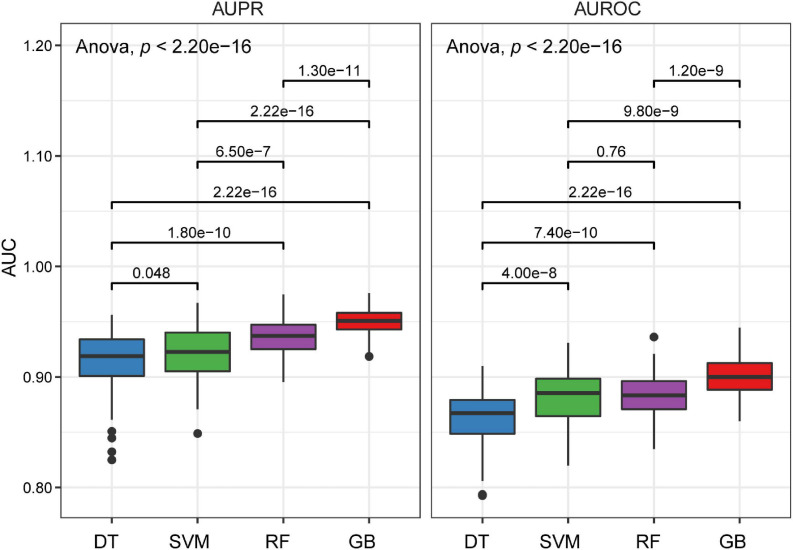
Boxplots of AUPRC and AUROC on the testing data for four different machine learning algorithms. *P* values were calculated through a one-way analysis of variance with Tukey’s *post hoc* test.

Next, we randomly divided the discovery dataset into two groups 100 times: training data (70%) and testing data (30%). Each time, four cutting-edge machine learning algorithms with the optimized parameters were used to develop models on the training data. Based on the obtained models, we used the testing data to assess the probability of EAS of the testing population, and ROC and PRC analyses were performed, followed by calculating the AUC values on the testing data. We compared the models and observed that DT was associated with significantly lower AUROC and AUPRC values, whereas the GB approach has higher AUROC and AUPRC values (Figure 2). Moreover, the two ensemble learning algorithms (RF and GB), especially GB, have lower variances compared to DT and SVM (Figure 2). Altogether, the GB algorithm outperformed the other machine learning algorithms in terms of the classification capacity of EAS.

### Predictive Model Construction and Validation

**FIGURE 3:**
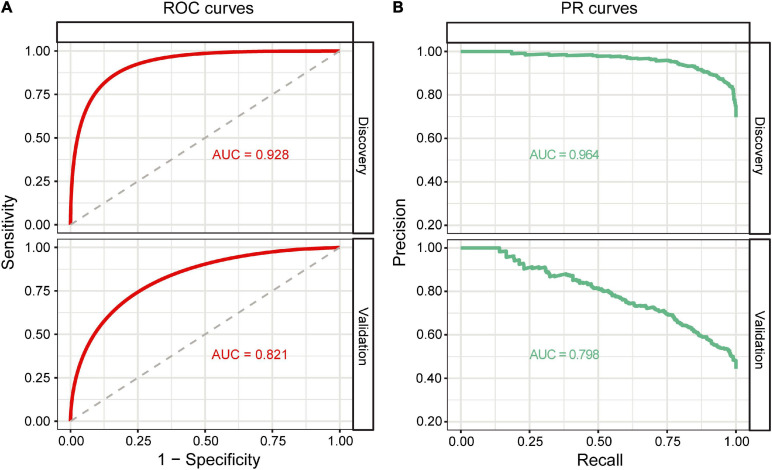
Classification performance of the GB model. **(A)** ROC curves of the GB model on the discovery and validation datasets. **(B)** PR curves of the GB model on the discovery and validation datasets.

Based on the GB algorithm, we finalized our EAS predictive model by training the GB model on the full discovery data with optimized parameters and calculated GB-based risk scores. In addition, we applied the GB model to the validation data from an independent Japanese cohort. Each predictor and other demographic information for the discovery and validation data are shown in Table 2. The AUROC and AUPRC values were assessed in both cohorts. The results showed high AUROC values of 0.928 and 0.821 and AUPRC values of 0.964 and 0.798 in the discovery and validation datasets, respectively, for the classification between non-EAS and EAS (Figures 3A,B).

**TABLE 2:** Comparison of clinical and demographical characteristics between the discovery and validation cohorts.

	Discovery set		Validation set		
	Non-EAS	EAS	Non-EAS	EAS	*P*
Total Num	230	530	507	405	
Age	48 ± 13	60 ± 9	48 ± 9	55 ± 9*	<0.001
Gender	–	–	–	–	0.003
Male	153 (66.5%)	304 (57.4%)	314 (61.9%)	278 (68.6%)	–
Female	77 (33.5%)	226 (42.6%)	193 (38.1%)	127 (31.4%)	–
SBP	115 ± 11	139 ± 19	114 ± 12	128 ± 15*	<0.001
DBP	70 ± 8	81 ± 11	72 ± 8^#^	81 ± 10	<0.001
BMI	24.16 ± 3.96	24.31 ± 3.42	22.96 ± 3.33^#^	23.34 ± 2.84*	<0.001
baPWV	1,219 ± 129	1,767 ± 305	1,259 ± 97^#^	1,612 ± 236*	<0.001

*Num, number; SBP, systolic blood pressure; DBP, diastolic blood pressure; BMI, body mass index; baPWV, brachial-ankle pulse wave velocity.*

#p < 0.05 nonVA in discovery set vs. nonVA in validation set; *p < 0.05 VA in discovery set vs. VA in validation set.

**FIGURE 4:**
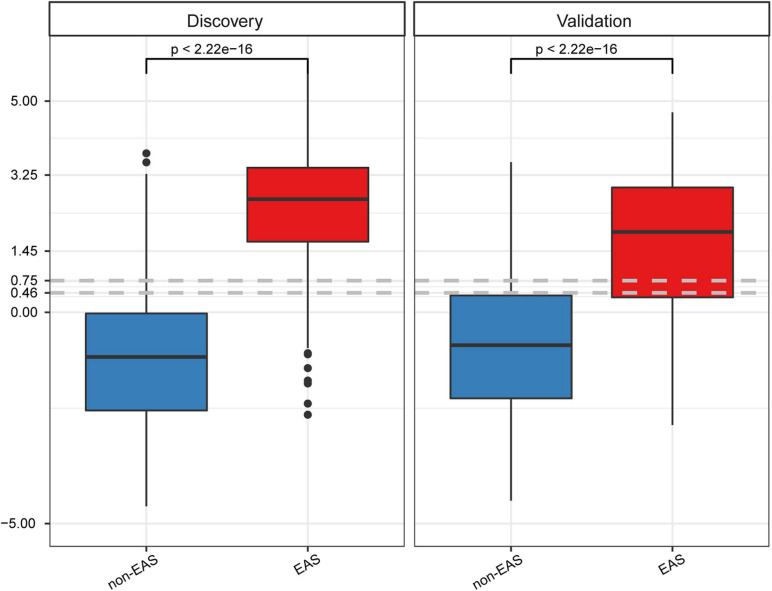
GB scores on the discovery and validation datasets between non-EAS and EAS. *P* values were calculated using Student’s *t*-tests.

To determine the best cutoff value of the GB model, Youden’s indexes were calculated in both cohorts. The cutoff value (0.75) built on the discovery cohort was higher than that (0.46) of the validation cohort (Figure 4), suggesting that the cutoff value derived from one cohort might not be ideal for other cohorts from different countries. Given that a cutoff value of 0.46 resulted in a better classification performance in both cohorts relative to a cutoff value of 0.75, which led to more false negative findings due to low sensitivity (0.677; Supplementary Table 6), we, therefore, selected 0.46 as a cutoff value for GB scores. The specificity/sensitivity in the discovery and validation cohorts at this cutoff value for the GB scoring system were 0.813/0.875 and 0.761/0.738, respectively (Supplementary Table 6).

### Web Tool Development

**FIGURE 5:**
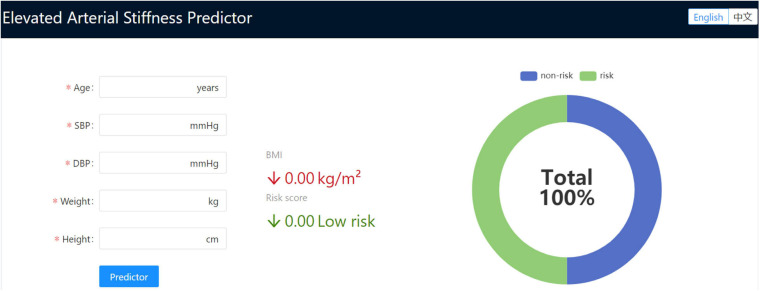
Screenshot of the web-based application (elevated arterial stiffness predictor).

To facilitate further study and use of this GB model for EAS prediction, we built a free and user-friendly online web-based tool (elevated arterial stiffness predictor:^3^). Figure 5 shows the user interface (UI) of the web tool. To use this web application, one only needs to input values for age, SBP, DBP, weight, and height, followed by clicking the “Predictor” button. Then, the UI will display the BMI and GB risk score value for this subject.

## Discussion

The increase in baPWV is considered a characteristic manifestation of EAS (Cunha et al., 2017). An increase of 1 m/s in baPWV will increase the mortality due to cardiovascular events, CVDs, and all-cause mortality by 12, 13, and 6%, respectively (Vlachopoulos et al., 2012). Thus, an affordable, reproducible, and accurate method for predicting EAS is desirable to support longitudinal surveillance and clinical decision making. In this study, we aim to construct a new EAS scoring system based on machine learning to identify EAS. To our knowledge, this is the first time that machine learning methods have been applied to develop an EAS predictor. Moreover, this model has been packaged into a user-friendly web application to encourage further study of its clinical utility.

Given that the clinical data we collected are relatively complete, including 99 features, we first narrowed down all features into 15 features based on the LASSO algorithm and 15 features based on the SVM-RFE algorithm. Four features (age, SBP, DBP, and BMI) from either the LASSO or SVM-RFE were further selected as the predictors in that they are easily accessible in clinical practice. Evidence suggests that the increase in age, SBP, and DBP are the important risk factors of artery stiffness (Papaioannou et al., 2019; Sang et al., 2020). Consistently, we observed a significant increase in age, SBP, and DBP in the EAS group compared to the non-EAS group. Furthermore, Baier et al. (2018) reported that age and SBP could explain 18% of the changes in PWV. Therefore, when predicting EAS and VA, age, SBP, and DBP are indispensable predictors. BMI was also determined as a diagnostic predictor of EAS by the SVM-RFE algorithm. Although no statistically significant difference in BMI between the EAS and non-EAS groups was observed, the multivariate logistic regression analysis with age, SBP, and DBP adjustments showed that BMI was an independent protective factor for EAS (*p* = 0.001, OR = 0.890; Supplementary Table 7). This result was similar to that of previous studies, which indicated that a high BMI was a protective factor for EAS and a higher BMI was associated with a lower baPWV (Lurbe et al., 2012; Huang et al., 2019; Yang et al., 2019).

Recently, the SAGE scoring system (including SBP, age, glycemia, and eGFR predictors) was established to predict EAS, which was an important step for the EAS surveillance and identification (Xaplanteris et al., 2019; Tomiyama et al., 2020). However, SAGE requires the predictors, i.e., glycemia and eGFR, to be obtained by an invasive blood test. Moreover, the LG-based SAGE system is not capable of obtaining interactions between predictors, which may affect the performance of SAGE. On the contrary, machine learning algorithms capable of capturing complicated interactions perform well in disease and prognosis prediction. For example, Alvin et al. pointed out that the predictive model based on machine learning could reliably identify patients who have high-risk diseases and increase the utilization of healthcare services (Rajkomar et al., 2019). David et al. used machine learning algorithms to improve the cardiovascular risk prediction of patients with end-stage renal disease on hemodialysis (de Gonzalo-Calvo et al., 2020). Michalis et al. also found that, based on machine learning algorithms, using volatile organic compounds in exhaled gas as predictors distinguishes lung cancer from other lung diseases or healthy individuals well (Zhang Z. et al., 2021). We used the four machine learning algorithms to develop the EAS predictive model. The results showed that all the models performed well with AUROC > 0.85 and AUPRC > 0.90, and particularly, GB outperformed other methods in terms of the AUROC, AUPRC, and variance. Owing to the limitations of the algorithm, DT constructs the model based on a single tree, and often suffers from overfitting (Katardjiev et al., 2019). Furthermore, if a certain correlation exists between the variables in the data, DT may cause a loss of associated information and reduction of accuracy. Thus, DT showed the relatively poor performance compared to other methods in this study. SVM maps data from low to high dimensional space using a kernel function to handle non-linearly separable data (Li et al., 2020). In the mapping process, if the kernel function does not discretize the data, that is, the data are sensitive to the kernel function, SVM may lead to a decrease in accuracy. However, ensemble learning algorithms such as RF and GB do not rely on a kernel function for data preprocessing, which integrate multiple prediction models that are trained on independent datasets and combined in a certain manner to make an overall prediction (Che et al., 2011). This yields more accurate results than those predicted by a single model. Therefore, these two methods performed better in this study. RF with a strong anti-interference ability can handle missing data especially in biomedical research. RF parallelly combines the results of multiple DTs to obtain the final model and does not further optimize the training results of different DTs, which may be the reason why the performance of RF is lower than that of GB. Unlike RF, which is based on bagging strategy and DT, GB is a combination of boosting strategy and DT. Boosting uses the residual value obtained in each iteration as the target value of the next iteration to further build the classification tree, whereas bagging parallelly trains multiple models based on training data randomly and independently sampled with replacement from the original dataset (Hall et al., 2011). GB keeps track of model’s errors, and assigns a higher weight to a good model. With the increase of number of iterations, the predictive ability of the GB model gradually improves and becomes stable. The advantages of the GB algorithm may be the reason why GB performed best in our dataset. Thus, we selected the GB model to predict EAS. The GB model showed good performance in the discovery and external verification datasets with AUROC and AUPRC values of 0.928/0.821 and 0.964/0.798, respectively. Compared to the SAGE, the GB scoring system not only has easier accessible predictors (age, SBP, DBP, and BMI vs. age, SBP, fasting glucose, and eGFR) but also higher AUROC values (0.928/0.821 vs. 0.85/0.77) in the discovery and external validation cohorts, respectively (Xaplanteris et al., 2019), further suggesting that the machine learning model outperforms the LG-based model.

Another point that needs to be discussed is the cutoff values of the GB model. Compared to the discovery cohort, there was a trend for lower GB scores in the EAS group of the validation cohort (Figure 4), which might be a result of the relatively higher age, SBP, and baPWV values in the EAS group of the discovery cohort than those of the validation cohort (Table 2). Differences in demographic and clinical characteristics may contribute to differences in the optional cutoff value, which prompted us to select a lower cutoff value to achieve a more rational classification performance. The first cutoff value (0.75) of the GB model was an optional trade-off point in the discovery cohort, whereas the second cutoff value (0.46) showed a better classification performance in the validation cohort. For the GB scoring system, we observed that the best trade-off point (0.75) for the discovery cohort showed biased classification in the validation cohort. After changing the trade-off point from 0.75 to 0.46, we observed better sensitivity (from 0.677 to 0.738; 6) without drastically decreasing the specificity (from 0.797 to 0.761; Supplementary Table 6). Thus, the demographic and clinical characteristics should be considered when determining the cutoff value.

Certain limitations of this study should be noted. First, the training sample size was limited. We plan to recruit more subjects from multiple center sites in the future to further increase the robustness of the model. Second, although this study was based on a Chinese cohort and validated using a Japanese cohort, prospective studies in different countries are required to further validate the results. Lastly, limitations in clinical data sharing infrastructure and mechanisms hinder further validation of cutting-edge machine learning methods (Geifman et al., 2015). We have packaged our GB model into a web-based application to encourage its dissemination for independent testing by other researchers.

In summary, we applied a cutting-edge machine learning method, GB, to establish an EAS scoring system for the identification of patients with EAS. We also validated the predictive performance of our GB model in an independent cohort from Japan. This GB model may help predict individual EAS risk and help clinicians manage patients with EAS.

## Data Availability Statement

The original contributions presented in the study are included in the article/Supplementary Material, further inquiries can be directed to the corresponding author/s.

## Ethics Statement

The studies involving human participants were reviewed and approved by Medical Faculty of Fujian Medical University Union Hospital Ethics Committee. The ethics committee waived the requirement of written informed consent for participation.

## Author Contributions

HH and LbL lead the study. QL, WX, and LpL performed the data analysis, implemented the methodology, and generated the web-based tool. LW, QY, LC, and JL collected the data and discussed the results. JF, WX, QL, and LpL prepared the original draft. HH, LL, WX, QL, and LpL reviewed and edited the final manuscript. All authors contributed to the article and approved the submitted version.

## Conflict of Interest

The authors declare that the research was conducted in the absence of any commercial or financial relationships that could be construed as a potential conflict of interest.

## Publisher’s Note

All claims expressed in this article are solely those of the authors and do not necessarily represent those of their affiliated organizations, or those of the publisher, the editors and the reviewers. Any product that may be evaluated in this article, or claim that may be made by its manufacturer, is not guaranteed or endorsed by the publisher.

## Footnotes

1
https://doi.org/10.5061/dryad.m484p

2
https://www.R-project.org

3
http://vascularagingpredictor.top/
