# Supplementary material for: Development and Validation of a Prediction Model for Elevated Arterial Stiffness in Chinese Patients With Diabetes Using Machine Learning
Source: Front Physiol. 2021 Aug 23;12:714195. doi: 10.3389/fphys.2021.714195 (PMC8419456; doi:10.3389/fphys.2021.714195)
Supplement: Supplementary file 1 [file Data_Sheet_1.docx]

Supplementary Material

# Supplementary Figures


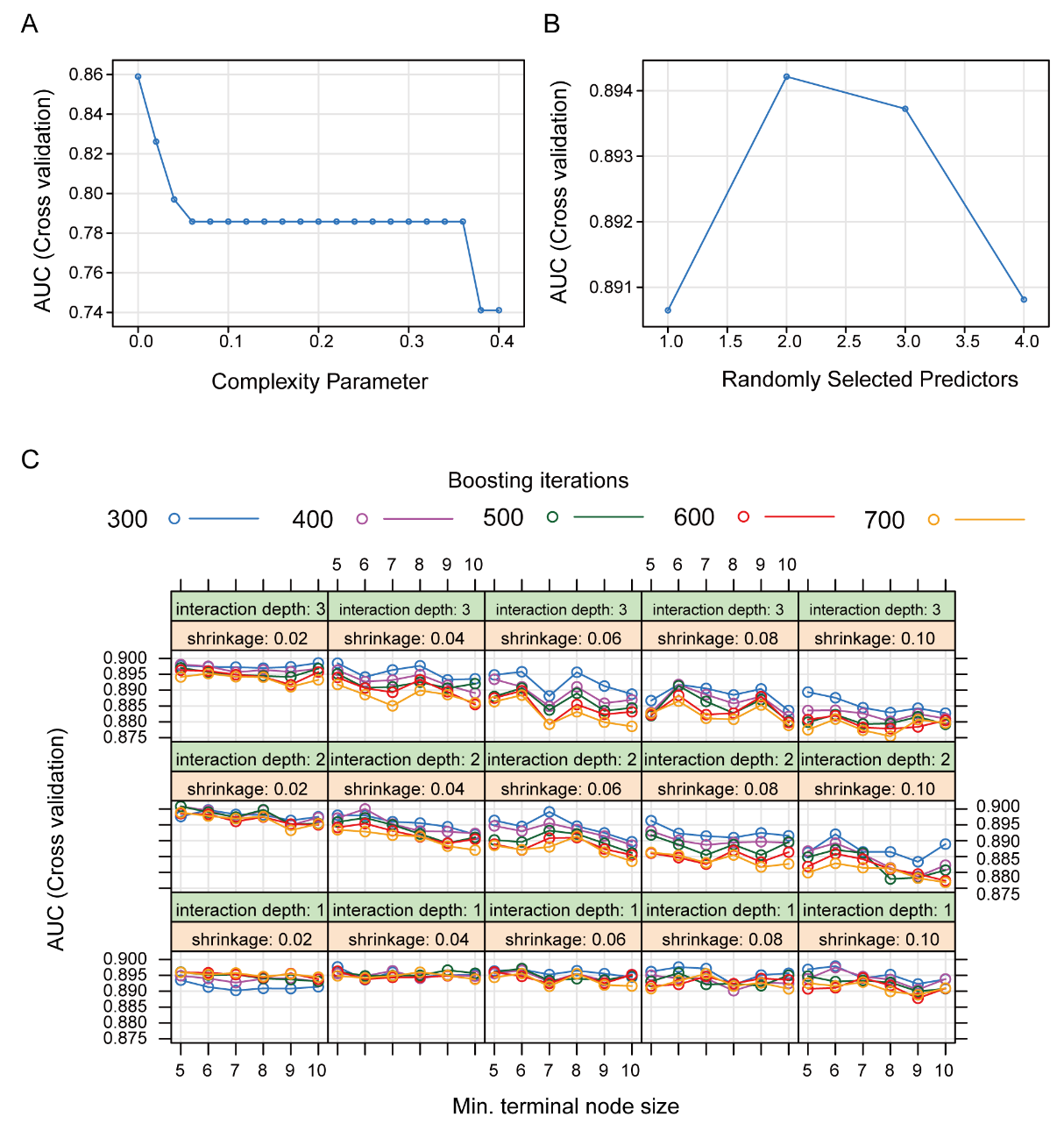


**Supplementary Figure 1.** Parameter tuning of models. (A) Optional complexity parameter of the DT model, (B) optional mtry parameter of the RF model, and (C) optional interaction.depth, n.tree, shrinkage, and n.minobsinnode parameters of the GB model were all selected based on a 10-fold cross-validation.

# Supplementary Tables

**2.1 Supplementary Table 1. Hemodynamic characteristics of the patients.**

|  | **Total** | **Non-EAS** | **EAS** | ***P*** |
| --- | --- | --- | --- | --- |
| *n* | 760 | 230 | 530 |  |
| PEP, ms | 113.43 ± 24.5 | 109.9 ± 24.22 | 114.96 ± 24.48 | < 0.001 |
| ET, ms | 289.68 ± 33.58 | 290.46 ± 31.07 | 289.35 ± 34.63 | 0.661 |
| ET/PEP | 2.55 ± 0.59 | 2.64 ± 0.61 | 2.50 ± 0.58 | 0.003 |
| HR, bpm | 73.51 ± 12.15 | 70.52 ± 11.64 | 74.81 ± 12.14 | < 0.001 |
| RbSBP, mmHg | 131.84 ± 20.1 | 116 ± 11.57 | 138.72 ± 19.11 | < 0.001 |
| LbSBP, mmHg | 131.2 ± 20.47 | 114.84 ± 11.87 | 138.3 ± 19.32 | < 0.001 |
| MeanSBP, mmHg | 131.52 ± 20.15 | 115.42 ± 11.45 | 138.51 ± 19.08 | < 0.001 |
| RaSBP, mmHg | 148.9 ± 26.5 | 129.28 ± 16.85 | 157.41 ± 25.39 | < 0.001 |
| LaSBP, mmHg | 148.98 ± 26.53 | 129.31 ± 16.93 | 157.52 ± 25.39 | < 0.001 |
| RbMAP, mmHg | 99.9 ± 15.72 | 87.28 ± 9.45 | 105.37 ± 14.72 | < 0.001 |
| LbMAP, mmHg | 99.15 ± 16.04 | 86.25 ± 10.05 | 104.75 ± 14.89 | < 0.001 |
| MeanMAP, mmHg | 99.53 ± 15.73 | 86.77 ± 9.48 | 105.06 ± 14.65 | < 0.001 |
| RaMAP, mmHg | 100.17 ± 15.69 | 88.69 ± 9.98 | 105.15 ± 15.1 | < 0.001 |
| LaMAP, mmHg | 100.41 ± 15.85 | 88.38 ± 10.33 | 105.62 ± 14.97 | < 0.001 |
| RbDBP, mmHg | 77.91 ± 11.12 | 70.31 ± 8.14 | 81.2 ± 10.62 | < 0.001 |
| LbDBP, mmHg | 77.56 ± 11.63 | 69.24 ± 8.47 | 81.17 ± 10.96 | < 0.001 |
| MeanDBP, mmHg | 77.73 ± 11.21 | 69.78 ± 8.03 | 81.19 ± 10.63 | < 0.001 |
| RaDBP, mmHg | 75.65 ± 11.13 | 68.61 ± 8.33 | 78.7 ± 10.8 | < 0.001 |
| LaDBP, mmHg | 75.41 ± 11.47 | 67.93 ± 8.78 | 78.66 ± 10.97 | < 0.001 |
| RbPP, mmHg | 53.94 ± 12.96 | 45.68 ± 7.93 | 57.52 ± 13.08 | < 0.001 |
| LbPP, mmHg | 53.64 ± 12.73 | 45.6 ± 8.01 | 57.13 ± 12.83 | < 0.001 |
| MeanPP, mmHg | 53.79 ± 12.68 | 45.64 ± 7.72 | 57.32 ± 12.78 | < 0.001 |
| RaPP, mmHg | 71.16 ± 19.76 | 59.22 ± 13.85 | 76.34 ± 19.71 | < 0.001 |
| LaPP, mmHg | 73.57 ± 19.63 | 61.38 ± 13.4 | 78.86 ± 19.56 | < 0.001 |
| RbUT, ms | 166.72 ± 40.25 | 165.84 ± 38.18 | 167.09 ± 41.14 | 0.694 |
| LbUT, ms | 170.18 ± 38.51 | 170.15 ± 36.84 | 170.2 ± 39.25 | 0.988 |
| RaUT, ms | 140.79 ± 19.36 | 143.03 ± 16.14 | 139.81 ± 20.54 | 0.021 |
| LaUT, ms | 140.78 ± 18.92 | 143.54 ± 15.95 | 139.58 ± 19.97 | 0.004 |
| LABI | 1.12 ± 0.09 | 1.1 ± 0.09 | 1.12 ± 0.09 | 0.008 |
| RABI | 1.12 ± 0.08 | 1.1 ± 0.08 | 1.12 ± 0.08 | 0.006 |
| LbaPWV, cm/s | 1610.46 ± 368.12 | 1226.7 ± 139.99 | 1776.99 ± 306.82 | < 0.001 |
| RbaPWV, cm/s | 1591.53 ± 369.38 | 1211.59 ± 136.69 | 1756.42 ± 312.53 | < 0.001 |
| MeanbaPWV,cm/s | 1601 ± 364.94 | 1219.15 ± 129.27 | 1766.7 ± 304.86 | < 0.001 |

**Abbreviations:** PEP, pre-ejection period; ET, ejection time; ET/PEP, systolic time intervals; HR, heart rate; RbSBP、LbSBP、RaSBP、LaSBP, systolic blood pressure of right brachial artery, left brachial artery, brachial artery, right ankle artery and left ankle artery, respectively; MeanSBP, average value of systolic blood pressure of right brachial artery and left brachial artery ; RbMAP、LbMAP、RaMAP、LaMAP, mean artery pressure of right brachial artery, left brachial artery, brachial artery, right ankle artery and left ankle artery, respectively; MeanMAP, average value of mean artery pressure of right brachial artery and left brachial artery ; RbDBP、LbDBP、RaDBP、LaDBP, diastole pressure of right brachial artery, left brachial artery, brachial artery, right ankle artery and left ankle artery, respectively; MeanDBP, average value of diastole pressure of right brachial artery and left brachial artery ; RbPP、LbPP、RaPP、LaPP, pulse pressure of right brachial artery, left brachial artery, brachial artery, right ankle artery and left ankle artery, respectively; MeanPP, average value of pulse pressure of right brachial artery and left brachial artery ; RbUT、LbUT、RaUT、LaUT, pulse upper time of right brachial artery, left brachial artery, brachial artery, right ankle artery and left ankle artery, respectively; LABI、RABI, left and right ankle brachial index, respectively; LbaPWV、RbaPWV、MeanbaPWV, left, right and average value of brachial ankle pulse wave velocity, respectively.

**2.2 Supplementary Table 2. Echocardiographic ultrasound characteristics of the patients.**

|  | **Total** | **Non-** **EAS** | **EAS** | ***P*** |
| --- | --- | --- | --- | --- |
| **Lved, mm** | 45.75 ± 4.54 | 45.85 ± 5.45 | 45.71 ± 4.18 | 0.296 |
| **Lves, mm** | 28.61 ± 3.47 | 28.75 ± 3.09 | 28.56 ± 3.6 | 0.605 |
| **Lad, mm** | 33.9 ± 4.91 | 32.56 ± 4.80 | 34.37 ± 4.88 | < 0.001 |
| **Ao, mm** | 29.37 ± 3.79 | 28.19 ± 4.00 | 29.79 ± 3.63 | < 0.001 |
| **Lvpw, mm** | 9.76 ± 1.28 | 9.28 ± 1.09 | 9.94 ± 1.3 | < 0.001 |
| **Ivs, mm** | 10.39 ± 1.57 | 9.72 ± 1.17 | 10.63 ± 1.62 | < 0.001 |
| **Rv, mm** | 20.15 ± 1.82 | 19.92 ± 1.91 | 20.23 ± 1.78 | 0.116 |
| **Rvod, mm** | 27.34 ± 3.37 | 26.62 ± 3.44 | 27.59 ± 3.31 | 0.030 |
| **Pa, mm** | 19.95 ± 1.89 | 19.76 ± 1.96 | 20.02 ± 1.86 | 0.214 |
| **E, m/s** | 0.72 ± 0.18 | 0.77 ± 0.18 | 0.70 ± 0.18 | 0.002 |
| **A, m/s** | 0.81 ± 0.2 | 0.68 ± 0.16 | 0.86 ± 0.19 | < 0.001 |
| **Hr, bpm** | 75.66 ± 12.39 | 73.37 ± 13.18 | 76.47 ± 12.01 | 0.026 |
| **Fs, %** | 37.46 ± 4.38 | 37.67 ± 3.97 | 37.38 ±4.51 | 0.535 |
| **Ef, %** | 67.33 ± 5.63 | 67.62 ± 4.93 | 67.23 ± 5.86 | 0.492 |
| **Sv, ml** | 65.62 ± 14.21 | 67.21 ± 14.86 | 65.06 ± 13.95 | 0.160 |
| **Co, L/min** | 4.87 ± 1.17 | 4.83 ± 1.23 | 4.88 ± 1.15 | 0.709 |
| **Ci, L/min/m^2^** | 2.83 ± 0.68 | 2.74 ± 0.66 | 2.86 ± 0.69 | 0.118 |
| **BSA, m^2^** | 1.71 ± 0.20 | 1.74 ± 0.21 | 1.69 ± 0.19 | 0.025 |
| **LVM, g** | 162.59 ± 42.04 | 152.42 ± 37.58 | 166.18 ± 42.99 | 0.002 |
| **LVMI, g/m^2^** | 95.03 ± 21.17 | 86.66 ± 16.28 | 97.99 ± 21.92 | < 0.001 |
| **E', m/s** | 0.07 ± 0.06 | 0.08 ± 0.03 | 0.06 ± 0.06 | 0.003 |
| **A', m/s** | 0.1 ± 0.05 | 0.09 ± 0.02 | 0.1 ± 0.05 | 0.018 |
| **RWT** | 0.44 ± 0.07 | 0.41 ± 0.05 | 0.45 ± 0.07 | < 0.001 |
| **E/E’** | 11.67 ± 3.86 | 9.72 ± 2.89 | 12.36 ± 3.93 | < 0.001 |
| **E/A** | 0.88 ± 0.32 | 1.11 ± 0.35 | 0.8 ± 0.26 | < 0.001 |
| **PASP, mmHg** | 26.26 ± 6.56 | 24.69 ± 5.91 | 26.83 ± 6.71 | 0.018 |

**Abbreviations:** Lved, left ventricular end diastolic diameter; Lves, left ventricular end systolic diameter; Lad, left atrium diameter; Ao, ascending aorta diameter; Lvpw, left ventricular posterior wall thickness; Ivs, interventricular septal thickness; Rv, right ventricular diameter; Rvod, right ventricular outflow tract diameter; Pa, pulmonary artery diameter; Hr, heart rates; Fs, fractional shortening; Ef, ejection fraction; Sv, stroke volume; Co, cardiac output; Ci, cardiac index; BSA, body surface area; LVM, left ventricular mass; LVMI, left ventricular mass index; RWT, relative wall thickness; PASP, pulmonary artery systolic pressure.

**2.3 Supplementary Table 3. Carotid artery ultrasound characteristics of the patients.**

|  | **Total** | **Non-** **EAS** | **EAS** | ***P*** |
| --- | --- | --- | --- | --- |
| LCCA, mm | 6.10 ± 0.80 | 5.87 ± 0.77 | 6.18 ± 0.8 | < 0.001 |
| RCCA, mm | 6.24 ± 0.88 | 5.99 ± 0.89 | 6.33 ± 0.85 | < 0.001 |
| MCCA, mm | 6.17 ± 0.77 | 5.93 ± 0.78 | 6.25 ± 0.75 | < 0.001 |
| cIMTT | 185 (40.39%) | 36 (29.27%) | 149 (44.48%) | 0.003 |
| LcIMT, mm | 0.86 ± 0.22 | 0.80 ± 0.22 | 0.88 ± 0.22 | 0.001 |
| RcIMT, mm | 0.88 ± 0.22 | 0.82 ± 0.2 | 0.90 ± 0.22 | < 0.001 |
| McIMT, mm | 0.87 ± 0.19 | 0.81 ± 0.17 | 0.89 ± 0.20 | < 0.001 |
| Plaque | 304 (66.38%) | 56 (45.53%) | 248 (74.03%) | < 0.001 |
| Numbers of plaque |  |  |  |  |
| 1 | 93 (30.59%) | 19 (33.93%) | 74 (29.84%) |  |
| ≥ 2 | 211 (69.41%) | 37 (66.07%) | 174 (70.16%) | 0.549 |
| Largest plaque area, mm^2^ | 28.03 ± 30.54 | 22.91 ± 12.94 | 29.24 ± 33.29 | 0.030 |
| RI | 164 (35.81%) | 39 (31.71%) | 125 (37.31%) | 0.267 |
| LRI | 0.76 ± 0.06 | 0.75 ± 0.06 | 0.76 ± 0.06 | 0.207 |
| RRI | 0.75 ± 0.06 | 0.75 ± 0.06 | 0.75 ± 0.06 | 0.199 |

**Abbreviations:**LCCA, left common carotid artery diameter; RCCA, right common carotid artery diameter; MCCA, average value of left and right common carotid artery diameter; cIMTT, carotid artery intima-media thickening; LcIMT, left carotid artery intima-media thickness; RcIMT, right carotid artery intima-media thickness; Plaque, plaque formation of carotid artery; RI, resistance index increased; LRI, resistance index of left common carotid artery; RRI, resistance index of right common carotid artery.

**2.4 Supplementary Table 4. Features with non-zero β coefficients in the LASSO regression model.**

|  | **β** | **OR** |
| --- | --- | --- |
| Constant | -8.985 | 0 |
| Age, years | 0.014 | 1.014 |
| γ-GT, IU/L | 0.000 | 1 |
| ALB, g/L | 0.009 | 1.009 |
| ET/PEP | -0.255 | 0.775 |
| MeanSBP, mmHg | 0.052 | 1.053 |
| MeanDBP, mmHg | 0.024 | 1.024 |
| A, m/s | 0.451 | 1.57 |
| Hr, bpm | 0.013 | 1.013 |
| BSA, m2 | -0.132 | 0.876 |
| A', m/s | 0.077 | 1.08 |
| E/E’ | 0.045 | 1.046 |
| E/A | -0.872 | 0.418 |
| Plaque | 0.600 | 1.822 |
| Neutrophils, X10^9/L | 0.141 | 1.151 |
| Nephropathy | 0.073 | 1.076 |

**Abbreviations:** γ-GT, γ-glutamyl transpeptidase; ALB, Albumin; ET/PEP, systolic time intervals; MeanSBP, average value of systolic blood pressure of right brachial artery and left brachial artery ; MeanDBP, average value of diastole pressure of right brachial artery and left brachial artery ; Plaque, plaque formation of carotid artery; Nephropathy, Diabetic nephropathy.

**2.5 Supplementary Table 5. Top 15 important features ranked based on the SVM-RFE analysis.**

| **Features** | **AvgRank** |
| --- | --- |
| MeanSBP, mmHg | 1 |
| MeanDBP, mmHg | 8.2 |
| MeanPP, mmHg | 10.2 |
| Lved, mm | 17 |
| Rv, mm | 18.4 |
| Ivs, mm | 18.8 |
| ET/PEP | 19.2 |
| Weitht, kg | 21 |
| Sv, ml | 21.8 |
| A', m/s | 21.8 |
| BMI, kg/m^2^ | 22.6 |
| UA, umol/L | 24.4 |
| Hr, bpm | 26.2 |
| TC, mmol/L | 26.4 |
| PDW, % | 29.4 |

**Abbreviations:** RV, right ventricular diameter; Ivs, interventricular septal thickness; ET/PEP, systolic time intervals; Sv, stroke volume; BMI, Body mass index; UA, Uric acid; Hr, heart rates; TC, Total cholesterol; PDW, Platelet volume distribution width.

**2.6 Supplementary Table 6. Specificity and sensitivity of the GB model in different cutoff points on the discovery and validation datasets.**

| **Data Set** | **Cutoff value** | **Specificity** | **Sensitivity** |
| --- | --- | --- | --- |
| **Discovery Set** | 0.75 | 0.857 | 0.851 |
|  | 0.46 | 0.813 | 0.875 |
| **Validation Set** | 0.75 | 0.797 | 0.677 |
|  | 0.46 | 0.761 | 0.738 |

**2.7 Supplementary Table 7.** **The multivariate logistic regression analysis.**

|  | ***P*** | **OR** | **95%CI(low to high)** |
| --- | --- | --- | --- |
| **Gender** | 0.900 | - | - |
| **Female** | - | 1.000 | - |
| **Male** | - | 0.969 | 0.596 to 1.576 |
| **Age, years** | 0.000 | 1.104 | 1.077 to 1.132 |
| **BMI, kg/m2** | 0.001 | 0.890 | 0.832 to 0.952 |
| **γ-GT, IU/L** | 0.518 | 1.000 | 0.998 to 1.001 |
| **eGFR, ml/min/1.73m2** | 0.665 | 0.998 | 0.988 to 1.007 |
| **SBP, mmHg** | 0.000 | 1.083 | 1.052 to 1.115 |
| **DBP, mmHg** | 0.000 | 1.081 | 1.036 to 1.128 |

**Abbreviations:** BMI, Body mass index; γ-GT, γ-glutamyl transpeptidase; eGFR, Estimated glomerular filtration rate; SBP, average value of systolic blood pressure of right brachial artery and left brachial artery; DBP, average value of diastole pressure of right brachial artery and left brachial artery.
